# Assessment of subpatent *Plasmodium* infection in northwestern Ethiopia

**DOI:** 10.1186/s12936-020-03177-w

**Published:** 2020-03-04

**Authors:** Ashenafi Assefa, Ahmed Ali Ahmed, Wakgari Deressa, G. Glenn Wilson, Amha Kebede, Hussein Mohammed, Maruon Sassine, Mebrahtom Haile, Dereje Dilu, Hiwot Teka, Matthew W. Murphy, Sheila Sergent, Eric Rogier, Zhou Zhiyong, Brian S. Wakeman, Chris Drakeley, Ya Ping Shi, Lorenz Von Seidlein, Jimee Hwang

**Affiliations:** 1 Ethiopian Public Health Institute, Arbegnoch Street, Mail Box: 19922, Addis Ababa, Ethiopia; 2 School of Public Health, Addis Ababa University, Addis Ababa, Ethiopia; 3 Department of Biology, University of Southern Denmark, 5230 Odense M, Denmark; 4 African Society for Laboratory Medicine, Addis Ababa, Ethiopia; 5 Malaria Branch, Division of Parasitic Diseases and Malaria, Centers for Disease Control and Prevention, Atlanta, GA USA; 6 Disease Prevention and Control Directorate, Federal Ministry of Health, Addis Ababa, Ethiopia; 7 U.S. President’s Malaria Initiative, United States Agency for International Development, Addis Ababa, Ethiopia; 8 Malaria Branch, Division of Parasitic Diseases and Malaria, Centers for Disease Control and Prevention, U.S. President’s Malaria Initiative, Addis Ababa, Ethiopia; 9 London School of Hygiene and Tropical Medicine, London, UK; 10 Mahidol Oxford Research Unit, Mahidol University, Bangkok, Thailand; 11 Malaria Branch, Division of Parasitic Diseases and Malaria, Centers for Disease Control and Prevention, U.S. President’s Malaria Initiative, Atlanta, GA USA

**Keywords:** Subpatent infections, Asymptomatic infections, Sub microscopic infections, Malaria, Ethiopia

## Abstract

**Background:**

Ethiopia has set a goal for malaria elimination by 2030. Low parasite density infections may go undetected by conventional diagnostic methods (microscopy and rapid diagnostic tests) and their contribution to malaria transmission varies by transmission settings. This study quantified the burden of subpatent infections from samples collected from three regions of northwest Ethiopia.

**Methods:**

Sub-samples of dried blood spots from the Ethiopian Malaria Indicator Survey 2015 (EMIS-2015) were tested and compared using microscopy, rapid diagnostic tests (RDTs), and nested polymerase chain reaction (nPCR) to determine the prevalence of subpatent infection. Paired seroprevalence results previously reported along with gender, age, and elevation of residence were explored as risk factors for *Plasmodium* infection.

**Results:**

Of the 2608 samples collected, the highest positive rate for *Plasmodium* infection was found with nPCR 3.3% (95% CI 2.7–4.1) compared with RDT 2.8% (95% CI 2.2–3.5) and microscopy 1.2% (95% CI 0.8–1.7). Of the nPCR positive cases, *Plasmodium falciparum* accounted for 3.1% (95% CI 2.5–3.8), *Plasmodium vivax* 0.4% (95% CI 0.2–0.7), mixed *P. falciparum* and *P. vivax* 0.1% (95% CI 0.0–0.4), and mixed *P. falciparum* and *Plasmodium malariae* 0.1% (95% CI 0.0–0.3). nPCR detected an additional 30 samples that had not been detected by conventional methods. The majority of the nPCR positive cases (61% (53/87)) were from the Benishangul-Gumuz Region. Malaria seropositivity had significant association with nPCR positivity [adjusted OR 10.0 (95% CI 3.2–29.4), P < 0.001].

**Conclusion:**

Using nPCR the detection rate of malaria parasites increased by nearly threefold over rates based on microscopy in samples collected during a national cross-sectional survey in 2015 in Ethiopia. Such subpatent infections might contribute to malaria transmission. In addition to strengthening routine surveillance systems, malaria programmes may need to consider low-density, subpatent infections in order to accelerate malaria elimination efforts.

## Background

The Global Technical Strategy for Malaria of the World Health Organization (WHO) calls for a world free of malaria [1]. To contribute to this global vision and encouraged by the substantial gains made in malaria control over the last two decades, Ethiopia has embarked on progressively eliminating malaria starting from low malaria transmission areas [2–6]. In the initial phase, 239 woredas (districts) were targeted for malaria elimination by the National Malaria Control and Elimination Programme of Ethiopia [5]. Preparations are underway to shift diagnosis and surveillance approaches from reducing malaria morbidity and mortality to detecting infections and measuring transmission in the selected woredas [2, 5].

Malaria elimination requires the detection and clearing of all *Plasmodium* infections. A relatively high prevalence of subpatent or low parasite density infections, that are often missed by conventional diagnostic methods (microscopy and rapid diagnostic tests) are being reported especially from low transmission settings [7–14]. Such asymptomatic, subpatent infections could be explained by acquired immunity in higher transmission settings [15]. Even in low transmission settings, asymptomatic and subpatent infections might play a role in transmission dynamics, hindering the progress of malaria elimination [14–19].

In Ethiopia, *Plasmodium falciparum* and *Plasmodium vivax* are the major reported malaria parasite species with rare reports of *Plasmodium malariae* and *Plasmodium ovale*. In 2017, out of a total confirmed malaria cases of 1,530,739, *P. falciparum* accounted for 1,059,847 cases and *P. vivax* for 470,892 cases [1, 2].

The current study assessed the magnitude of subpatent infections in northwestern Ethiopia, using samples collected in 2015 from a nationwide household survey of malaria endemic areas.

## Methods

### Study area

**Fig. 1:**
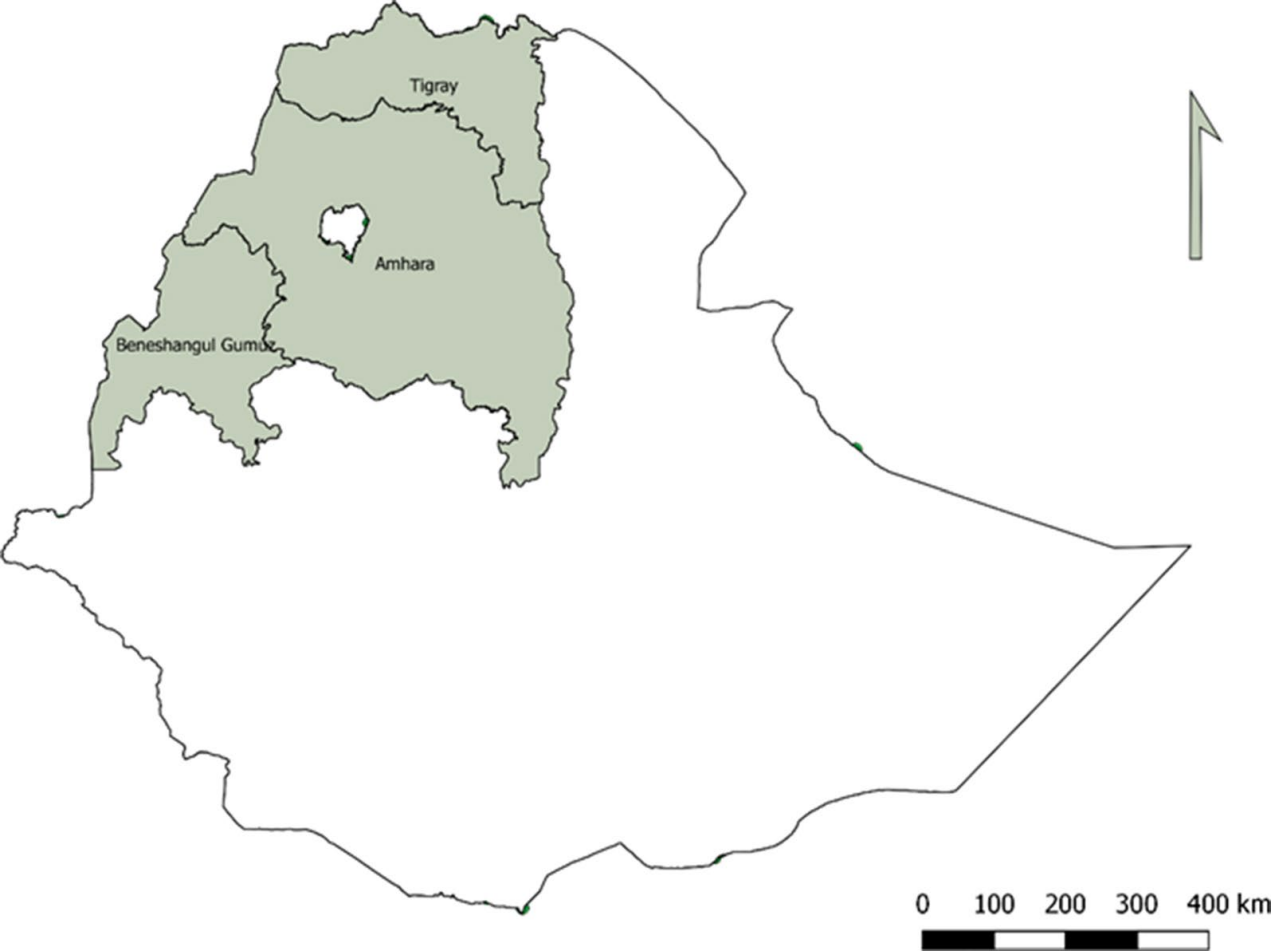
Ethiopia and location of the three sample collection regions (shaded grey): Tigray, Amhara and Benishangul-Gumuz

The study used Dried blood spot (DBS) samples collected as part of the National Ethiopian Malaria Indicator Survey conducted in 2015 (EMIS-2015) [20]. The survey was conducted during the peak of the malaria transmission season between September and December, 2015. Sample collection during EMIS-2015 was limited to areas below 2500 m elevation, with 85% of samples collected from areas below 2000 m elevation and the remaining 15% from areas between 2000 and 2500 m elevation. For the present study, samples were selected purposefully from the Amhara, Benishangul-Gumuz and Tigray Regions (Fig. 1). From the EMIS-2015 results, rapid diagnostic test (RDT) prevalence ranged from 10.4% in Benishangul-Gumuz, 1.9% in Tigray, to 1.1% in Amhara. A separate study [21] also found this geographical area to represent a range of malaria transmission from very low to moderate based on seroprevalence. Participants tested by malaria microscopy and RDT that provided DBS samples were selected from the EMIS 2015 samples repository at the Ethiopian Public Health Institute (EPHI) in Addis Ababa, Ethiopia.

### Study population and sample collection

EMIS-2015 was a cross-sectional survey, which used a two-stage cluster sampling methodology [20]. Enumeration Areas (EA), the sampling unit, were selected proportional to population size and 25 households were selected by simple random sampling from each EA. Demographic, socioeconomic, malaria prevention, and malariometric data were also collected in the selected households. All children under 5 years of age in selected households and persons of all ages in every 4th household were eligible for biological sample collection and testing [20]. Whole blood from a single finger prick from consenting individuals (with or without fever) was collected for *Plasmodium* infection identification by RDT and microscopy, haemoglobin testing (Hemocue Hb 201+, Hemocue AB, Ängelholm, Sweden) and for collection of DBS samples. Whatman 903 (GE Healthcare, Pittsburgh, PA) filter paper cards were used for DBS collection. These were air dried, individually packed in a plastic bag together with a desiccant and stored at − 20 °C at EPHI before they were sent to the U.S. Centers for Disease Control and Prevention (CDC) in, Atlanta, Georgia, for processing.

### *Plasmodium* infection exposure

Serology methods and results for these samples have been previously reported [21]. Briefly, DBS elutions were assayed using bead-based multiplex assays for IgG antibodies for six.

*Plasmodium* antigens: four human malaria species-specific merozoite surface protein-1 19 kD antigens (MSP-1) and Apical membrane antigen-1 (AMA-1) for *P. falciparum* and *P. vivax*.

### Parasite identification

#### Rapid diagnostic tests

The CareStart™ Malaria HRP2/pLDH (Pf/PAN) Combo RDT were used to detect malaria in the field. CareStart™ tests for histidine-rich protein 2 (HRP2) to detect *P. falciparum* and pan-*Plasmodium* lactose dehydrogenase (LDH) for *P. falciparum*, *P. vivax*, *P. ovale* and *P. malariae*.

#### Microscopy

Thick and thin blood smears were made on the same slide, air-dried, and transported to EPHI. The slides were stained with 3% Giemsa for 10 min and screened for the presence of *plasmodia*l infections. Microscopists were WHO certified and were blinded to the RDT and survey results. A slide was classified as negative if no *Plasmodium* asexual forms or gametocytes were found after viewing 100 fields. Any positive slide was confirmed by two other microscopists.

#### Polymerase chain reaction

**Fig. 2:**
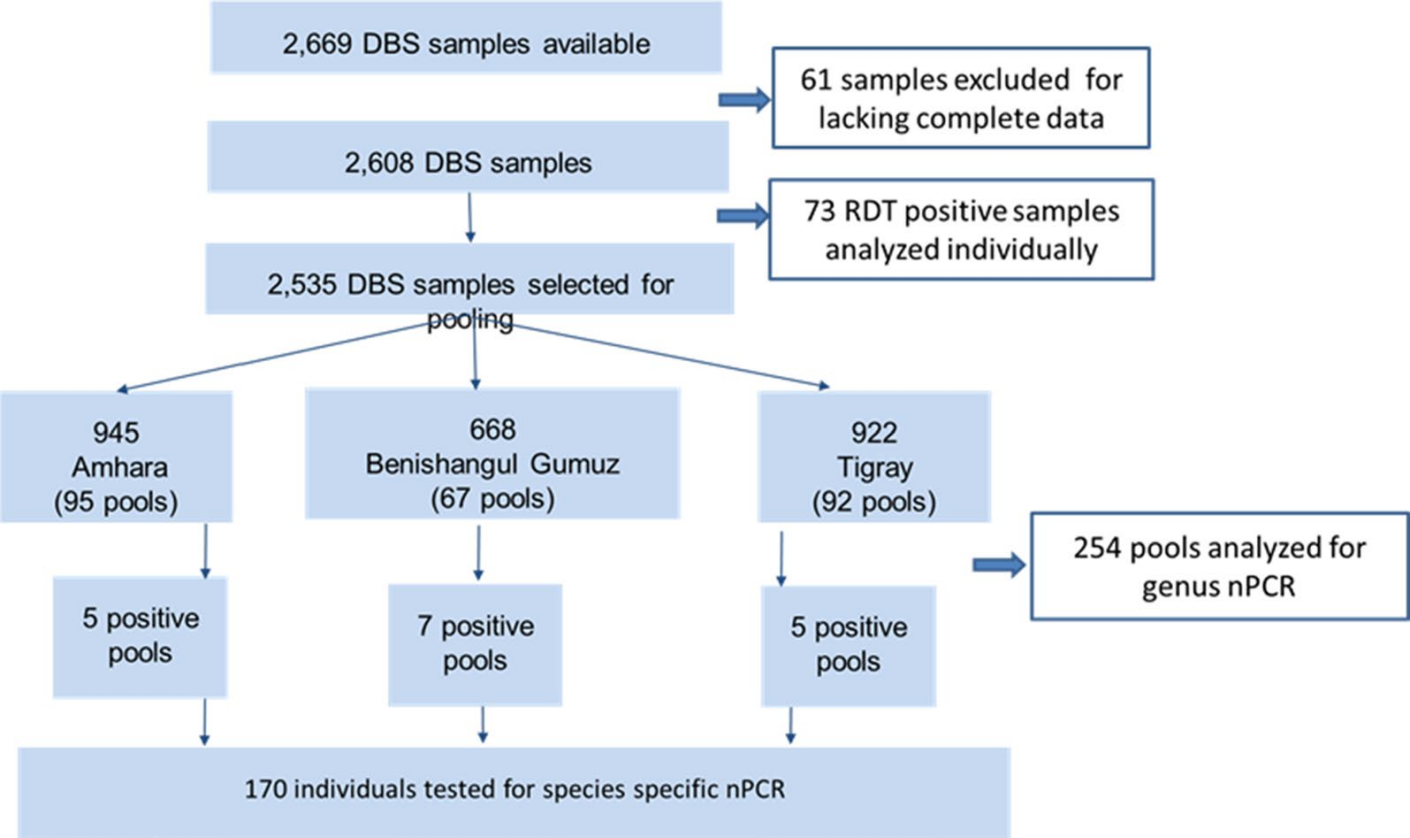
Flow chart showing sampling and pooling analysis procedures

The DBSs were shipped to CDC at ambient temperature. A total of 2533 RDT-negative and 2 samples without RDT results available from the three regions were pooled and analysed (Fig. 2). An additional 73 RDT-positive samples were analysed and confirmed by PCR individually.

Two-step pooling was used to estimate the prevalence of malaria in the RDT-negative samples, as previously described by Zhou et al. [23]. A pilot was carried out to test for the effect of age (pooled by less than or equal to five and above 5 years of age) and different numbers of sample pooling (five or ten samples per pool), but no major differences by age or pooling size were found. Subsequently, individual samples from the same region were randomized into pools of ten samples.

##### DNA extraction

A 6-mm disk was punched out from each DBS card and ten disks pooled for DNA extraction. Pooled DBSs were extracted with QIAamp DNA Mini kit as per the manufacturer’s protocol (QIAGEN, Valencia, CA) with slight modification. Samples were left for an overnight incubation in ATL buffer plus Proteinase K to ensure proper lysis during pooling, and the DNA was then extracted following a normal extraction protocol. The purified DNA was tested using nested genus PCR (nPCR). For any pool with a positive PCR result, individual DBSs were extracted following the same protocol as for the pooled samples and species-specific PCR assays were performed.

##### nPCR detection for 18S ribosome RNA (rRNA)

Purified DNA templates were used for amplification of the 18S rRNA gene subunit using a modified method as previously described [23]. Briefly, the 25 μL PCR reaction contained 1× PCR Master Mix (Promega, Madison, WI) and 100 nM of each PCR primer. For each primary PCR reaction, 5 μL of DNA were used. The reactions were performed under the conditions of initial denaturation at 95 °C for 5 min, 25 cycles at 95 °C for 1 min, annealing at 58 °C for 2 min, and final extension at 72 °C for 2 min, followed by a final extension at 72 °C for 10 min. Species PCR was performed as per primary PCR except for use of 35 cycles and 2.5 μL primary PCR product per reaction. Genus PCR was performed as per species PCR except annealing temperature was set at 64 °C. The amplified DNA products were stained with GelRed (Biotium, Hayward, CA), separated using 2% agarose gel electrophoresis and visualized under UV illumination.

### Data analysis

Data were analysed using Stata 13 (Stata Corp. 2015. Stata Statistical Software: Release 14. College Station, TX: Stata Corp LP, USA). Descriptive statistics with 95% confidence interval were estimated for demographic characteristics. Multiple logistic regression was used to assess the association of risk factors (gender, age, elevation, bed net use and seropositivity) with nPCR parasitaemia results. Percent relative difference and concordance for microscopy and RDTs were calculated against PCR results as the reference standard. Values were considered significant at P < 0.05. A Venn diagram was constructed using STATA to visualize the relationship amongst the four diagnostic methods: microscopy, RDTs, nPCR and serology. QGIS 2.18 was used to construct a map of the study area.

## Results

### Study population

Of the 2669 samples available from the three regions, 2535 samples were pooled for PCR analysis and 73 tested individually: 945 from Amhara, 668 from Benishangul-Gumuz and 922 from Tigray (Fig. 2). A total of 2608 samples had matched assay and survey data, comprising of 2533 RDT-negative, 73 RDT-positive and 2 without RDT results. About 53% (95% CI 50.4–54.2) of the samples tested were from females and 44% (95% CI 42.0–45.8) from children under 5 years of age (Table 1). The mean participant age from the samples was 16.1 years (95% CI 15.4–17.0) (range: 1–88 years). About 80% of the data were collected from areas below 2000 m in elevation. Of the 1354 samples with a paired seroprevalence result, almost half [51.3% (95% CI 48.6–53.9)] were seropositive of which 41.3% (95% CI 38.7–43.9) of the samples were seropositive for *P. falciparum* (MSP-1 or AMA-1 antigen responses) and 34.0% (95% CI 31.6–36.6) seropositive for *P. vivax (*MSP-1 or AMA-1). Additionally, 7.0% (95% CI 5.7–8.4) and 4.9% (95% CI 3.8–6.2) of participants were seropositive for *P. malariae* and *P. ovale* MSP-1 antigen responses, respectively. Overall, 59.6% (95% CI 57.3–61.9) of respondents providing samples reported having slept under a bed net in the previous night (Table 1).

**Table 1:** Characteristics of survey samples from Tigray, Amhara and Benishangul-Gumuz Regions of Ethiopia, 2015

Characteristics	Number	Proportion (%) (95% CI)
Sex	N = 2608	
Female	1364	52.3 (50.4–54.2)
Male	1244	47.7 (45.8–49.6)
Age	N = 2608	
< 5	1145	43.6 (42.0–45.8)
5–14 years	508	19.5 (18.0–21.0)
15–24 years	325	12.5 (11.2–13.8)
25–49 years	460	17.6 (16.2–19.1)
> 50 years	170	6.5 (5.6–7.5)
Elevation	N = 2608	
< 2000 m	2134	81.8 (80.3–83.3)
≥ 2000 m	474	18.2 (16.7–19.7)
Use of bed nets	N = 1798	
Yes	1072	59.6 (57.3–61.9)
No	726	40.4 (38.1–42.7)
Seropositivity	N = 1354	
*P. falciparum* (MSP-1 or AMA-1)	559	41.3 (38.7–43.9)
*P. vivax* (MSP-1 or AMA-1)	461	34.0 (31.6–36.6)
*P. malariae* (MSP-1)	94	7.0 (5.7–8.4)
*P. ovale* (MSP-1)	66	4.9 (3.8–6.2)
Any *Plasmodium* (MSP-1 or AMA-1)	694	51.3 (48.6–53.9)

### Prevalence of malaria infection

**Fig. 3:**
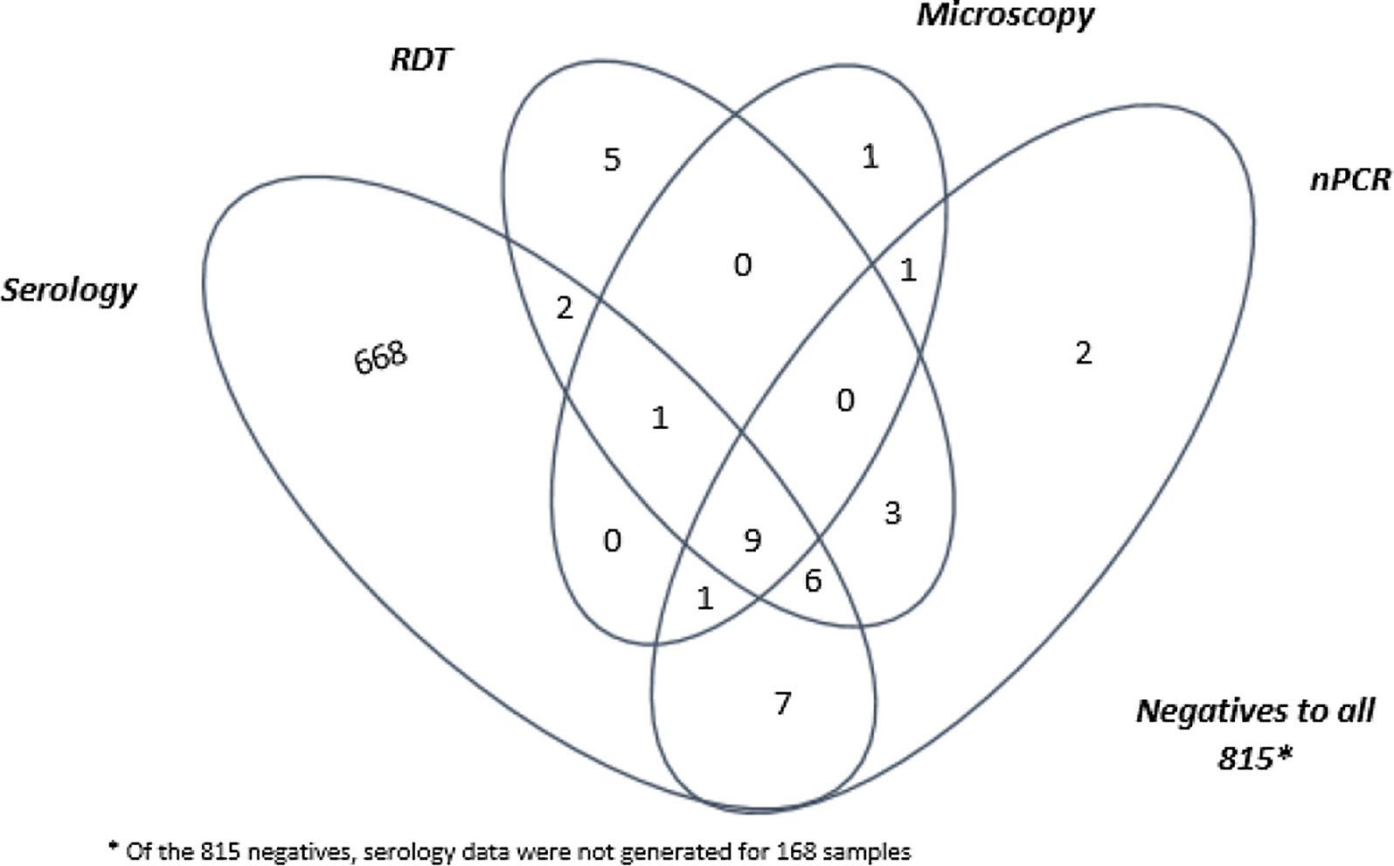
Venn diagram showing the relationships and overlap among serology (IgG MSP-1 or AMA-1), Rapid Diagnostic Tests (RDTs), Microscopy and nested Polymerase Chain Reaction (nPCR) methods, in 1522 samples with complete results collected from Tigray, Amhara and Benishangul-Gumuz Regions, Ethiopia as part of the Malaria Indicator Survey, 2015

The detection of *Plasmodium* infection varied by diagnostic methods: microscopy, RDT and nPCR (Table 2). Of the 2608 samples tested 2.8% (95% CI 2.2–3.5) were found to be positive for *Plasmodium* using RDTs, comprising of 1.9% (95% CI 1.5–2.5) due to *P. falciparum* (only HRP2 positive), 0.4% (95% CI 0.2–0.7) due to *P. falciparum* infection with or without other *Plasmodium* species (HRP2 and panLDH positive) and 0.5% (95% CI 0.3–0.5) were *P. vivax, P. ovale* or *P. malariae* infection (only panLDH positive). Using microscopy, 1.2% (95% CI 0.8–1.7) of the samples were found to be positive for *Plasmodium*, of which 1.0% (95% CI 0.6–1.4) were due to *P. falciparum* infection, 0.2% (95% CI 0.0–0.4) were due to *P. vivax* infection and 0.08% (95% CI 0.0–0.3) were due to mixed *P. falciparum* and *P. vivax* infection. RDT positivity was over twofold higher than microscopy positivity (Table 2). Figure 3 shows the relationships and overlap among serology, RDTs, microscopy and nPCR methods.

**Table 2:** Variation in malaria positivity of samples between RDT, microscopy and nPCR in the study area (Tigray, Amhara and Benishangul-Gumuz Regions), 2015

Diagnostic methods	Species diagnosed	Number of positives	Percent positivity (95% CI)	Percent relative change from PCR
RDT (n = 2606)	*P. falciparum* (HRP2+)	50	1.9 (1.5–2.5)	− 37.5
	*P. vivax*/*P. malariae*/*P. ovale* (panLDH+)	12	0.5 (0.3–0.8)	0.0
	*P. falciparum* or mixed (HRP2 and panLDH+)	11	0.4 (0.2–0.7)	N/A
	Total *Plasmodium* positive	73	2.8 (2.2–3.5)	− 16.1
Microscopy (n = 2605)	*P. falciparum*	25	1.0 (0.6—1.4)	− 68.8
	*P. vivax*	4	0.2 (0.0–0.4)	− 60.0
	Mixed *P. falciparum *+* P. vivax*	2	0.08 (0.0–.03)	− 33.3
	Total *Plasmodium* positive	31	1.2 (0.8–1.7)	− 64.4
nPCR (n = 2608)	*P. falciparum* monoinfection	80	3.1 (2.5–3.8)	
	*P. vivax* monoinfection	10	0.4 (0.2–0.7)	
	Mixed *P. falciparum* + *P. vivax*	3	0.1 (0.0–0.4)	
	Mixed *P. falciparum* + *P. malariae*	2	0.07 (0.0–0.3)	
	Total *Plasmodium* positive	87	3.3 (2.7–4.1)

Malaria DNA screening by nPCR detected 3.3% (95% CI 2.7–4.1) positivity for any malaria species. Of this, *P. falciparum* accounted for 3.1% (95% CI 2.5–3.8) of infection, *P. vivax* accounted for 0.4% (95% CI 0.2–0.7) of infection, mixed *P. falciparum* and *P. vivax* 0.1% (95% CI 0.0–0.4), and mixed *P. falciparum* and *P. malariae* 0.1% (95% CI 0.0–0.3). PCR detected 64.4% and 16.1% more positives compared to microscopy and RDT, respectively (P < 0.05). In addition, PCR detected two *P. malariae* cases mixed with *P. falciparum* cases. By RDT, these were both appropriately positive for HRP2 and panLDH antigens. Using microscopy, one of the *P. malariae* positive infection was missed and the other was identified as mixed infection. No *P. ovale* infection was identified by any diagnostic method (Table 2). Seropositive samples had a very high likelihood of being screened positive for malaria DNA [adjusted OR 10.0 (95% CI 3.2–29.4; P < 0.001)]. While living in lower elevation areas (< 2000 m), sex, age group, or bed net use were not associated with PCR positivity in the study samples (Table 3).

**Table 3:** Association between nPCR positivity and sample characteristics (sex, age, elevation, bed net use and seropositivity)

	PCR positive			
	Unadjusted OR (95% CI)	P-value	Adjusted OR (95% CI)	P-value
Sex				
Female	Ref		Ref	
Male	1.3 (0.8–2.0)	0.231	0.8 (0.5–1.2)	0.21
Age intervals (years)				
< 5	Ref		Ref	
5–14	1.2 (0.5–2.9)	0.758	1.3 (0.5–3.4)	0.535
15–24	1.0 (0.3–3.1)	0.974	1.3 (0.4–3.6)	0.702
25–49	0.5 (0.2–1.9)	0.343	0.7 (0.2–3.9)	0.528
≥ 50	0.4 (0.1–3.2)	0.407	0.5 (0.1–3.9)	0.512
Elevation (m)				
> 2000	Ref		Ref	
< 2000	7.2 (1.0–53.5)	0.052	5.0 (0.1–53.7)	0.118
Use of bed net (N = 1798)				
No	Ref		Ref	
Yes	0.7 (0.4–1.3)	0.267	0.8 (0.4–1.3)	0.345
Seropositivity				
No	Ref		Ref	
Yes	5.9 (2.0–17.1)	0.001	10.0 (3.2–29.4)	< 0.001

PCR identified 30 additional samples as positive that were negative by RDT (Table 4). Of these, 25 were due to *P. falciparum* infection, five were due to *P. vivax* infection, and two were due to a mixed infection of *P. falciparum* and *P. malariae*. Half of the additional positive samples (14 of 30), and mixed *P. falciparum* and *P. malariae* cases were identified from samples collected in Benishangul-Gumuz, a region of higher malaria transmission in Ethiopia.

**Table 4:** Malaria prevalence by nPCR from RDT-negative samples by region, 2015 EMIS

Region	RDT negative samples	PCR-positive samples				
		*P. falciparum*	*P. vivax*	*P. malariae*	*P. ovale*	Total positive, % (n)
Benishangul- Gumuz	668	12	2	2^a^	0	2.1% (14)
Amhara	944	8	2	0	0	1.1% (10)
Tigray	921	5	1	0	0	0.7% (6)
Total	2533	25	5	2^a^	0	1.2% (30)

73 samples that were RDT positive and 2 samples from Amhara and Tigray each not tested by RDT were excluded

^a^Mixed with *P. falciparum*

The percent concordance of RDTs and microscopy with nPCR detection on sample positivity varied between the three regions (Table 5). Overall, 80.8% (59/73) of the samples identified as positive by RDTs and 83.9% by microscopy were confirmed positive by nPCR. The highest agreement with nPCR data for both RDTs (90.7%) and microscopy (86.7%) was observed in the Benishangul-Gumuz samples where *P. falciparum* is predominant. In the Amhara and Tigray regions agreement with nPCR diagnosis was lower at 64.3% and 68.8%, respectively.

**Table 5:** Concordance of RDTs and slide positive samples compared to nPCR by region, 2015 EMIS

Region	Percent concordant, % (n/N)	
	RDT versus PCR	Slide versus PCR
Benishangul-Gumuz	90.7% (39/43)	86.7% (13/15)
Amhara	64.3% (9/14)	77.8% (9/7)
Tigray	68.8% (11/16)	71.4% (7/5)
Total	80.8% (59/73)	83.9% (26/31)

## Discussion

In this study, results of microscopy, RDT and nPCR detection methods were used to examine the prevalence of *Plasmodium* infection in malaria endemic settings in northwest Ethiopia. Nested PCR identified 30 additional positive malaria infections that were missed by RDTs in the field, and thus not provided treatment at the time of the survey. The prevalence reported by nPCR was 3.3%, 16.1% higher than that detected by RDTs and 64.4% higher than from microscopy. More than 60% of the nPCR positive cases were identified from Benishangul-Gumuz Region, which is one of the higher malaria transmission region in Ethiopia [5]. The region showed a similar high prevalence of malaria by microscopy and RDTs in this study and in a separate serology study of the same samples [21].

The study compared microscopy and RDT detection methods with a pooling nPCR approach that can detect parasite density as low as 0.1–10 parasites/µl of blood [22–26]. Close agreement was observed between both RDT and microscopy results compared to PCR with 80.8% and 83.9% concordance, respectively. Microscopy, considered the gold standard for malaria diagnosis [26], has a variable limit of detection (50–100 parasite/µl of blood) [26–28], depending on the skill of the technician and the reagents used [26–28]. As per the national malaria diagnosis and treatment guideline [29], Ethiopia uses microscopy in health centres and hospitals and RDTs in health posts (primary health care units). In the current study with slide preparation and RDTs conducted under field conditions where test requirements may not be optimal, high variability was observed in the results across the three methods. This discordance becomes disproportionately higher in these low transmission settings with very few positive samples. The strong association between seropositivity and PCR positivity suggests a role for using multiplex seroprevalence results to more rapidly and inexpensively identify hotspots or confirm lack of *Plasmodium* infection in areas reporting low incidence.

Although the lower RDT positivity is likely due to low-density infections, the possibility of hrp2/3 gene deleted *P. falciparum* infections has been raised in Ethiopia with alarming reports originating from Eritrea [29–33]. Although HRP2 and HRP3 deletions were reported recently from a study in Amhara [34], a study of larger geographic scope is currently ongoing in Ethiopia (Sindew Mekasha, EPHI, personal communication).

Several studies have reported asymptomatic *Plasmodium* infections in low transmission settings using PCR from a range of countries [7–15, 18, 22, 35, 36]. The terms asymptomatic, submicroscopic, and subpatent are often used interchangeably to describe a malaria infection that cannot be detected by conventional methods (microscopy and RDTs) but can be detected by more sensitive methods such as PCR. The prevalence of subpatent infection in low transmission setting has ranged from 0.003 to 44%, depending on the sensitivity of the tools used and the sample collection area [36]. Studies in Iran [37] and Sri Lanka [38] reported zero prevalence by the more sensitive methods, indicative of no local transmission and confirmation of malaria elimination. Golassa et al. [14] and Tadesse et al. [15] reported PCR prevalence ranging from 1.7 to 5.8% in southwest Ethiopia, comparable to the 3.3% prevalence reported in the current study.

Subpatent infections, despite the low parasite density, could be infectious to mosquitoes [15, 19, 39]. Studies in The Gambia [40], Thailand [35], Peru [41] and Ethiopia [17] showed even low-density, asymptomatic infections could be infectious to mosquitoes, which may pose a potential, unidentified reservoir for malaria transmission. A recent review [19] summarized that lower density of parasites were seen in low transmission compared to high transmission settings and argued that subpatent infections contribute to the infectious reservoir, could be long-lasting, and predictive of future periods of patent infections.

Several new tools are being developed with higher limits of parasite detection in field settings, such as, ultrasensitive RDTs and loop-mediated isothermal amplification (LAMP) [35, 42]. The current study used a sample pooling methodology that decreases cost and time and is applicable to screening large number of samples, particularly from low transmission settings [22, 43]. The low blood volume eluted from the DBSs in the current study may limit parasite detection as the volume of blood analysed is critical in determining its limit of detection [44, 45]. Although more sensitive molecular methods have been developed [46], the cost and feasibility of the tests in field settings could limit their wide-scale adoption and uptake in resource-limited countries.

## Conclusion

The current study reports the presence of subpatent infection in the samples collected as part of a national cross-sectional malaria survey in 2015. Using nPCR, the detection of malaria parasites increased by nearly threefold over rates compared to microscopy. Subpatent infections could potentially be infective to mosquitoes contributing to ongoing malaria transmission. Malaria elimination efforts may need to consider low-density, subpatent infections in managing sources of infectious reservoirs or hotspots, in addition to strengthening routine surveillance and response systems.

## Data Availability

The datasets used and/or analysed during the current study are available from the corresponding author on reasonable request.

## Abbreviations

AMA-1: Apical membrane antigen-1
CDC: Centers for Disease Control and Prevention
DBS: Dried blood spot
EA: Enumeration Areas
EMIS: Ethiopian Malaria Indicator Survey
EPHI: Ethiopian Public Health Institute
HRP2: Histidine-rich protein 2
MSP-1: Merozoite surface protein-1
nPCR: Nested polymerase chain reaction
pLDH: *Plasmodium* lactose dehydrogenase
RDTs: Rapid diagnostic tests
rRNA: Ribosome RNA
WHO: World Health Organization
